# Novel Echocardiographic Index for Risk Stratification of Ventricular Arrhythmias and Mortality Based on Right Ventricular Function

**DOI:** 10.1002/joa3.70244

**Published:** 2026-01-08

**Authors:** Toshinori Chiba, Takatsugu Kajiyama, Yusuke Kondo, Hiroyuki Takaoka, Noriko Suzuki‐Eguchi, Masahiro Nakano, Miyo Nakano, Satoko Ryuzaki, Yukiko Takanashi, Yuya Komai, Yusei Nishikawa, Yoshio Kobayashi

**Affiliations:** ^1^ Department of Advanced Cardiorhythm Therapeutics Chiba University Graduate School of Medicine Chiba Japan; ^2^ Deutsches Herzzentrum der Charité, Campus Mitte, Department of Cardiology Berlin Germany; ^3^ Department of Advanced Arrhythmia Bioengineering Chiba University Graduate School of Medicine Chiba Japan; ^4^ Department of Cardiovascular Medicine Chiba University Graduate School of Medicine Chiba Japan

**Keywords:** heart failure, ICD therapy, right ventricular dysfunction, sudden cardiac death

## Abstract

**Background:**

Right ventricular (RV) dysfunction is independently predictive of sudden cardiac death. This study aimed to compare the performance of different risk stratification methods for death and appropriate implantable cardioverter‐defibrillator (ICD) therapy using echocardiography and cardiac magnetic resonance imaging (CMR) to quantify RV function.

**Methods:**

Consecutive patients undergoing ICD implantations who had completed both preprocedural echocardiography and CMR were retrospectively enrolled. Patients with channelopathies or arrhythmogenic right ventricular disease were excluded. The RV fractional area change (RVFAC) and estimated pulmonary artery pressure (EPAP) were calculated from echocardiography. The contraction pressure index (CPI) was defined as the quotient of the RVFAC divided by the EPAP. Both metrics were used to predict the composite endpoint of death and an appropriate ICD therapy. RV dysfunction was defined by either RVFAC < 35% or RV ejection fraction (RVEF) < 45%.

**Results:**

In total, 88 patients (60.4 ± 14.7 years, 61 males) including 15 with ischemic cardiomyopathy were retrospectively enrolled. Forty‐two patients received ICDs as secondary prevention. The mean RVFAC, CPI, and RVEF were 35.9% ± 9.22%, 1.4% ± 0.7%/mmHg, and 39.5% ± 14.4%, respectively. Regarding the composite endpoint, the best cut‐off value of the CPI was 1.59 (specificity 0.45, sensitivity 0.96, ROC‐AUC 0.68). The hazard ratio of a low RVFAC was 3.28 (95% CI: 1.39–7.77, *p* = 0.007, concordance = 0.622), a low CPI, 14.2 (95% CI: 1.91–104.9, *p* = 0.010, *c* = 0.665), and a low RVEF, 3.44 (95% CI: 1.17–10.1, *p* = 0.003, *c* = 0.620).

**Conclusion:**

Both CMR‐derived RVEF and the echocardiographic CPI predicted appropriate ICD therapy and death. The CPI may provide superior risk stratification.

## Graphical abstract

Patients undergoing ICD implantation with both preprocedural echocardiography and cardiac magnetic resonance (CMR) were retrospectively analyzed to assess right ventricular (RV) function. Echocardiographic RV fractional area change (RVFAC) and the contraction pressure index (CPI), as well as CMR‐derived RV ejection fraction (RVEF), predicted appropriate ICD therapy and death, with CPI demonstrating superior prognostic performance.

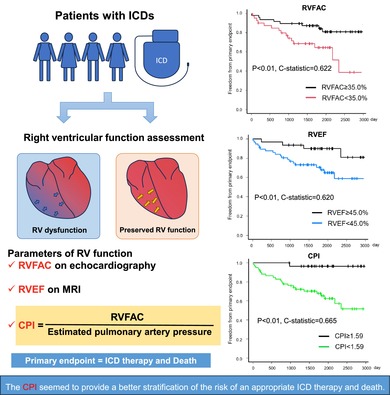

## 1 Introduction

The incidence of sudden cardiac death (SCD) reportedly ranges from 30 to 100 per 10 000 people worldwide, and it has been reported that the incidence of SCD increases with aging [1, 2]. Automated external defibrillators and implantable cardiac defibrillators have rapidly prevailed and have contributed to an improvement in the prognosis. However, the incidence of ventricular fibrillation (VF) as the initial rhythm in SCD remained constant in the United States from 2007 to 2015. Similarly, VF and ventricular tachycardia (VT) as causes of out‐of‐hospital cardiac arrests remained stable at 25% in Germany from 2006 to 2020 [3, 4]. The efficient stratification of the risk of SCD is still a major concern in public health. The AHA/ACC/HRS/ESC guidelines adopt left ventricular (LV) dysfunction as a key criterion for considering implantable cardioverter defibrillator (ICD) implantations as primary prevention [5, 6]. Some studies describing ICD implantations in patients with LV dysfunction have prevented SCD‐associated VF and VT by delivering shocks and anti‐tachycardia pacing (ATP) therapy [7, 8]. On the other hand, Stecker et al. reported that 60% of SCD cases have a left ventricular ejection fraction (LVEF) > 35%, and 48% of cases with SCD have a normal LV systolic function [9]. Thus, the stratification by only the LVEF might be inadequate, and additional risk factors of SCD have to be sought.

Recently, several studies have shown a significant relationship between SCD and right ventricular (RV) dysfunction. Pandat et al. reported an association between a reduced right ventricular fractional area change (RVFAC) and SCD [10]. In addition, they reported that RV dysfunction defined by an RVFAC < 35% measured on transthoracic echocardiography (TTE) is associated with an increased risk of SCD independent of the LVEF. We also reported that patients with ICDs and a reduced RVFAC experience significantly more frequent appropriate ICD therapies than patients whose RVFAC is normal [11]. On the other hand, for the evaluation of right ventricular (RV) function, the right ventricular ejection fraction (RVEF) obtained by cardiac magnetic resonance imaging (CMR) is considered the gold standard. No study has yet determined which modality is superior for stratifying the risk of an appropriate ICD therapy and death. The purpose of this study was to compare the RVFAC on echocardiography and RVEF on CMR in the context of a prognostic prediction.

## 2 Methods

### 2.1 Study Population

This study was a single‐center retrospective cohort study. We enrolled consecutive patients who received initial ICD implantations in our institution from 2012 to 2020. The patient characteristics including the age, sex, height, weight, and presence of diabetes, lung disease, underlying disease, prevention, and antiarrhythmic drugs were collected retrospectively. We excluded patients with arrhythmogenic right ventricular cardiomyopathy (ARVC), Brugada syndrome, and long QT syndrome from this study because the patients with ARVC generally have a decreased RV systolic function and an arrhythmogenic RV myocardium. We exclusively enrolled those who underwent both TTE and CMR before the index implantation. All patients included in the study underwent both TTE and CMR under clinically stable conditions and not during episodes of acute decompensated heart failure, prior to the index ICD implantation. All patients gave their written consent prior to the procedure. The present study was approved by the institutional domestic ethical committee, which waived the requirement for informed consent considering the study's observational design. The study complied with the Declaration of Helsinki.

### 2.2 Follow‐Up and Endpoint

The patients were followed up in our clinic for a device check‐up every 6 months after their initial implantation of the ICD. When a remote monitoring system detected alerts including sustained VT, VF, delivery of ATP, and shock therapy, the patient was called to confirm the status and to present to ambulatory care if needed. We defined an appropriate ICD therapy as an ICD shock and ATP therapy for VT/VF. The primary endpoint was defined as a composite of all‐cause death and an appropriate ICD therapy.

### 2.3 Echocardiogram Measurements

All patients underwent TTE prior to the index implantation of an ICD using a Vivid E9 console (GE Vingmed Ultrasound AS, Horten, Norway) and EchoPAC PC BT 13 software (GE HealthCare, Horten, Norway). The right ventricular function was retrospectively evaluated in addition to the standard left ventricular measurements by one physician and one echocardiologist separately. In brief, on the four‐chamber view, the endocardial contour was traced to calculate the RV end‐diastolic area (RVEDA), RV end‐systolic area (RVESA), and RV basal diameter. The RVFAC was calculated as the difference between the RVEDA and RVESA, divided by the RVEDA [12]. The LVEF was measured using the standard Simpson biplane method in the four‐ and two‐chamber views. We adopted the mean RVFAC of those two RVFAC values in the statistics. RV dysfunction was defined as RVFAC < 35% according to the previous study. The estimated pulmonary artery pressure (EPAP) was calculated using the tricuspid regurgitation (TR) peak gradient (TRPG), inferior vena cava (IVC) diameter, and its variation. According to the recommendations for cardiac chamber quantification by echocardiography in adults [13], we calculated the estimated right atrial pressure (ERAP) and divided it into 3 pressure grades (an IVC diameter < 21 mm and collapses of > 50% with a sniff‐defined normal pressure of 3 mmHg, IVC diameter > 21 mm, and collapses of < 50% with a sniff‐defined high pressure of 15 mmHg, and the other cases were defined as an intermediate pressure of 8 mmHg). We excluded patients whose EPAP could not be calculated due to missing data or the absence of tricuspid regurgitation. The EPAP was calculated as the sum total of the TRPG and ERAP. We defined the contraction pressure index (CPI) as the RVFAC divided by the EPAP.

### 2.4 Cardiac Magnetic Resonance Measurements

We retrospectively analyzed the RV function on the preoperative CMR images while the RVEF was semiautomatically calculated on MR Multi‐Chamber Wall Motion Tracking software (Canon, Japan). Briefly, the right ventricular endocardium was traced automatically on the software and then the RVEF was calculated. When the endocardium could not be accurately traced automatically, the examiner manually adjusted the traced endocardium. RV dysfunction was defined as RVEF < 45% according to the previous study.

### 2.5 Statistics

The continuous variables are reported as the means ± standard deviations and were compared using a Student's *t*‐test. The differences between the proportions were compared using Chi‐squared tests. Differences in the distribution of TR grades among groups were evaluated using the Cochran–Armitage trend test for ordinal data. The history of catheter ablation for ventricular arrhythmias prior to ICD implantation or before the first appropriate therapy was analyzed for its impact on the outcome. A receiver‐operator characteristics (ROC) curve was analyzed to determine the best cut‐off (BCO) value of the baseline CPI to predict the primary endpoint. The BCO values were determined according to the maximum sum of the sensitivity and specificity. The correlation of the objects was analyzed to calculate the Pearson Correlation Coefficient. A Kaplan–Meier curve was described for the primary endpoint. In the univariate analysis and multivariate analyses, a Cox hazard regression was used to test the related factors for RV dysfunction, and we compared the *c*‐statistic of each factor to validate the model's accuracy. We considered *p* values of ≤ 0.05 as statistically significant. When there were patients with missing data for a specific analysis, we excluded them from the specific analysis. All statistics were analyzed using R software version 4.0.3 (R Foundation for Statistical Computing, Vienna, Austria).

## 3 Results

### 3.1 Patient Characteristics and Endpoints

In total, 88 patients (60.4 ± 14.7 years, 61 males) were enrolled in the present study. The baseline characteristics of the patients are shown in Table 1. Forty‐two patients (47.7%) received an ICD as secondary prevention. Approximately half of the patients were taking oral class 3 antiarrhythmic agents. Fifteen patients (17.0%) harbored ischemic cardiomyopathy (ICM). VT of unknown etiology (UVT) was defined as cases that had a normal left ventricular function and no findings on the CMR, cardiac computed tomography, or endomyocardial biopsy.

**TABLE 1:** Characteristics of the total patients and patients divided into two groups according to the composite endpoint of an appropriate ICD therapy and death.

*n* = 88	Total	ICD therapy or death (−) (*n* = 64)	ICD therapy or death (+) (*n* = 24)	*p*
Male, *n* (%)	61 (69.3)	44 (68.8)	17 (70.8)	1.000
Age, years	60.4 ± 14.7	59.0 ± 14.4	64.0 ± 15.0	0.157
BMI, kg/m^2^	23.6 ± 3.85	24.0 ± 3.92	22.5 ± 3.48	0.114
Secondary prophylaxis, *n* (%)	42 (47.7)	28 (43.8)	14 (58.3)	0.241
Βeta blocker, *n* (%)	83 (94.3)	59 (92.2)	24 (100)	0.317
Class 3 antiarrhythmic drug, *n* (%)	42 (47.7)	22 (34.4)	20 (83.3)	< 0.001
Hypertension, *n* (%)	36 (40.9)	25 (39.1)	11 (45.8)	0.630
Diabetes, *n* (%)	16 (18.2)	11 (17.2)	5 (20.8)	0.759
Chronic kidney disease, *n* (%)	37 (42.0)	24 (37.5)	13 (54.2)	0.225
Atrial fibrillation, *n* (%)	32 (36.4)	20 (31.2)	12 (50.0)	0.136
Lung disease, *n* (%)	12 (13.6)	10 (15.6)	2 (8.3)	0.500
Right CAD, *n* (%)	9 (10.2)	8 (12.5)	1 (4.2)	0.434
RBBB, *n* (%)	19 (21.6)	13 (20.3)	6 (25.0)	0.772
S‐ICD, *n* (%)	5 (5.7)	3 (4.7)	2 (8.3)	0.611
Inappropriate ICD therapy, *n* (%)	7 (8.0)	4 (6.2)	3 (12.5)	0.385

*Note:* Data are presented as the mean standard ± deviation or *n* (%).

Abbreviations: BMI, body mass index; CAD, coronary artery disease; RBBB, right bundle branch block; S‐ICD, subcutaneous implantable cardioverter defibrillator.

### 3.2 Measurements on TTE and CMR

**FIGURE 1:**
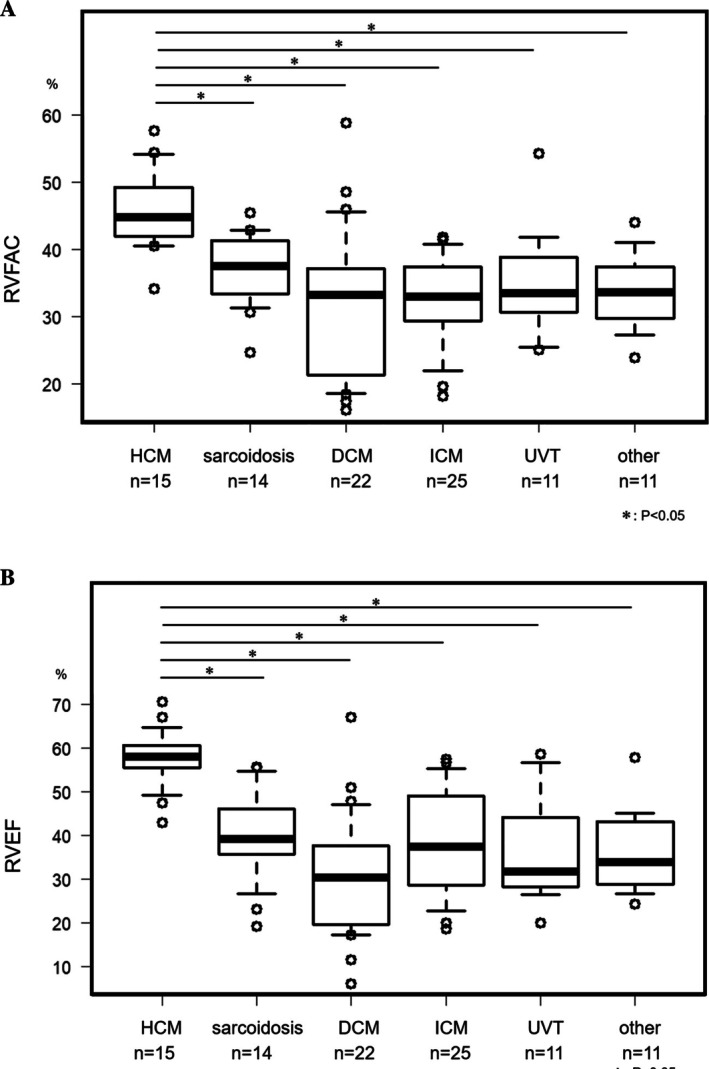
(A) Hypertrophic cardiomyopathy (HCM) patients had a significantly higher RVFAC than the others, but there was no difference between HCM and sarcoidosis, and there was no difference in the background diseases except for HCM. (B) Difference in the RVEF between the background diseases. Only HCM had a higher RVEF than the other diseases, and the CPI between the background diseases also exhibited this trend. DCM, dilated cardiomyopathy; ICM, ischemic cardiomyopathy; UVT, ventricular tachycardia of unknown etiology.

The baseline echocardiographic data is shown in Table 2. The mean LVEF, RVFAC, and RVEF were 40.2% ± 17.2%, 35.9% ± 9.22%, and 39.5% ± 14.4%, respectively. The mean EPAP and CPI were 29.3 ± 9.44 mmHg and 1.39% ± 0.67%/mmHg. There was a fair correlation between the RVFAC and LVEF (correlation coefficient = 0.42, *p* < 0.001). There was also a fair correlation between the RVEF and LVEF (correlation coefficient = 0.55, *p* < 0.001). There was also a fair correlation between the CPI and LVEF (correlation coefficient = 0.46, *p* < 0.001). There was a moderate correlation between the RVFAC and RVEF (correlation coefficient = 0.65, *p* < 0.001). Although the LVEF in the low RVFAC and low RVEF groups was significantly lower than in the other group, the EPAP calculated from the TTE was significantly higher in the low RVFAC and low RVEF groups (*p* = 0.019, *p* = 0.001). On the other hand, there was a significant difference between the background diseases with respect to their RV function. Figure 1A shows that patients with hypertrophic cardiomyopathy (HCM) significantly had a higher RVFAC than the others. Figure 1B shows the RVEF difference between the background diseases. Only HCM had a higher RVEF than the other diseases, and the CPI between the background diseases also exhibited this trend.

**TABLE 2:** Results of transthoracic echocardiography according to each indicator of RV dysfunction.

	Overall (*n* = 88)	RVFAC ≥ 35% (*n* = 48)	RVFAC < 35% (*n* = 40)	*p*	RVEF ≥ 45% (*n* = 31)	RVEF < 45% (*n* = 57)	*p*	CPI ≥ 1.59 (*n* = 29)	CPI < 1.59 (*n* = 59)	*p*
LVEF, %	40.2 ± 17.2	44.5 ± 17.5	35.1 ± 15.5	0.010	50.3 ± 18.0	34.7 ± 14.0	< 0.001	48.2 ± 18.0	36.3 ± 15.	0.002
LVDd, mm	57.7 ± 10.5	54.5 ± 9.1	61.6 ± 10.8	0.001	52.6 ± 8.94	60.5 ± 10.3	0.001	54.4 ± 10.2	59.3 ± 10.3	0.037
LVEDV, mm	163.4 ± 79.3	143 ± 64.5	187 ± 88.7	0.010	124.7 ± 64.8	185.2 ± 78.9	< 0.001	139 ± 68.3	175.8 ± 82.2	0.042
LAD, mm	44.7 ± 7.93	45.3 ± 7.66	44.0 ± 8.27	0.439	45.4 ± 8.17	44.4 ± 7.85	0.583	43.8 ± 7.9	45.2 ± 7.9	0.428
RVFAC, %	35.9 ± 9.22	42.3 ± 5.98	28.1 ± 5.60	< 0.001	42.2 ± 7.0	32.4 ± 8.37	< 0.001	41.4 ± 11.4	29.7 ± 8.2	< 0.001
EPAP, mmHg	29.3 ± 9.44	27.2 ± 7.60	31.9 ± 10.8	0.019	24.9 ± 6.50	31.7 ± 9.98	0.001	21.6 ± 5.2	33.2 ± 8.8	< 0.001
TRPG, mmHg	25.4 ± 9.06	23.5 ± 7.33	27.7 ± 10.4	0.029	21.26 ± 6.69	27.7 ± 9.42	0.001	18.2 ± 5.14	29.0 ± 8.48	< 0.001
TR grade	I: 51 (58.0%), II: 29 (33.0%), III: 8 (9.1%)	I: 32 (66.7%), II: 14 (29.2%), III: 2 (4.2%)	I: 19 (47.5%), II: 15 (37.5%), III: 6 (15.0%)	0.208	I: 23 (74.2%), II: 7 (22.6%), III: 1 (3.2%)	I: 28 (49.1%), II: 22 (38.6%), III: 7 (12.3%)	0.063	I: 24 (82.8%), II: 5 (17.2%), III: 0 (0.0%)	I: 27 (45.8%), II: 24 (40.7%), III: 8 (13.6%)	0.008
RVEF, %	39.5 ± 14.4	46.4 ± 13.2	31.2 ± 10.9	< 0.001	55.7 ± 6.41	30.7 ± 8.61	< 0.001	49.5 ± 12.3	34.6 ± 12.7	< 0.001

*Note:* Data are presented as the mean ± standard deviation or *n* (%). TR (tricuspid regurgitation) grade was classified as follows: I = mild, II = moderate, and III = severe.

Abbreviations: EPAP, estimated pulmonary arterial pressure; LAD, left atrial diameter; LVDd, left ventricular end‐diastolic diameter; LVEDV, left ventricular end‐diastolic volume; LVEF, left ventricular ejection fraction; RVEF, right ventricular ejection fraction; RVFAC, right ventricular fractional area change; TRPG, tricuspid regurgitation pressure gradient.

### 3.3 Follow‐Up and the Primary Endpoint

The mean follow‐up duration was 1864 ± 674 days. A total of 17 initial appropriate ICD therapies were observed, with nine cases of ATP therapy and eight of shock therapy. Six patients died from cardiovascular diseases, whereas one patient died from a non‐cardiovascular disease during the follow‐up period.

We found that 13 patients underwent catheter ablation: five before ICD implantation and eight after implantation. In the preceding‐ablation group, 1 out of 5 patients received appropriate therapy. In the postimplantation ablation group, five appropriate therapies were observed, and the ablation procedures were performed after those first therapies. The remaining three patients underwent ablation procedures but did not experience any appropriate therapy. Therefore, it is reasonable to consider including the eight cases in which the ablation procedure was performed not before the first therapy. Univariate analysis revealed no significant effect of ablation procedures on the outcome (HR 0.57 [0.075–4.32], *p* = 0.586).

**FIGURE 2:**
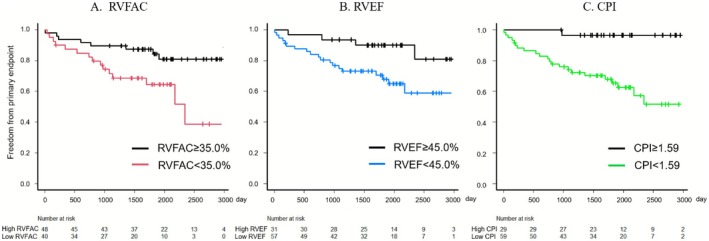
Kaplan–Meier curve for the primary endpoint consisting of an appropriate ICD therapy and death, individually stratified by the right ventricular fractional area change (RVFAC), right ventricular ejection fraction (RVEF), and the contraction‐pressure index (CPI), which was defined as the quotient of the RVFAC divided by the estimated pulmonary arterial pressure. (A) Stratification using the best cut‐off value of the RVFAC. (B) Stratification using the best cut‐off value of the RVEF. (C) Stratification using the best cut‐off value of the CPI. The primary endpoint significantly increased in the low RVFAC, RVEF, and CPI groups. RV dysfunction was significantly associated with an appropriate ICD therapy and death.

The RVFAC and CPI in the patients with the primary endpoint were significantly lower than those in the patients without the primary endpoint (RVFAC: 32.1% vs. 37.3%, *p* = 0.017; CPI: 1.10 vs. 1.50%/mmHg, *p* = 0.012); however, there was no difference in the RVEF. Regarding the composite endpoints as an initial appropriate ICD therapy and death, the BCO value of the CPI was 1.59%/mmHg (specificity 0.45, sensitivity 0.96, ROC‐AUC 0.68). In the entire cohort, 40 patients (45.5%) and 57 patients (64.7%) had RV dysfunction according to the criterion of the RVFAC and RVEF, respectively. There were 59 patients (67.0%) with a low CPI in the cohort. Regarding the primary endpoint, Figure 2 shows the Kaplan–Meier curves for survival according to each indicator. A low RVFAC, low RVEF, and low CPI were significantly associated with a higher incidence of the primary endpoint. The hazard ratio of the primary endpoint in the low RVFAC group was 3.28 (95% CI: 1.39–7.77, *p* = 0.007), RVEF group, 3.44 (95% CI: 1.17–10.1, *p* = 0.003), and low CPI group, 14.2 (95% CI: 1.91–104.9, *p* = 0.010), as shown in Table 3.

**TABLE 3:** Cox hazard regression analysis for the primary endpoint.

	Univariate analysis			*c*‐statistics	SE	Multivariate analysis		
	HR	95% CI	*p*			HR	95% CI	*p*
Age	1.03	1.00–1.06	0.093					
Male	1.25	0.44–3.59	0.679					
Secondary prophylaxis	3.08	1.07–8.86	0.037			1.48	0.62–3.53	0.375
LVEF < 35%	0.95	0.42–2.13	0.897			0.53	0.23–1.24	0.142
Class 3 AAD	11.0	2.49–48.7	0.002			7.70	2.52–23.6	< 0.001
S‐ICD	1.89	0.44–8.12	0.393					
VT ablation	0.57	0.01–4.32	0.586					
RVFAC < 35%	3.28	1.39–7.77	0.007	0.622	0.052			
RVEF < 45%	3.44	1.17–10.1	0.003	0.620	0.041			
CPI < 1.59	14.2	1.91–104.9	0.010	0.665	0.032	13.5	1.80–100.6	0.011

Abbreviations: AAD, anti‐arrhythmic drug; CI, confidence interval; CPI, contraction pressure index; HR, hazard ratio; LVEF, left ventricular ejection fraction; RVEF, right ventricular ejection fraction; RVFAC, right ventricular fractional area change; SE, standard error; S‐ICD, subcutaneous implantable cardioverter defibrillator; VT, ventricular tachycardia.

In the univariate analysis for the primary endpoint, RV dysfunction defined as an RVFAC < 35%, RVEF < 45%, and CPI < 1.59 was a predictor of a primary endpoint event, as shown in Table 3. Furthermore, the *c*‐statistic of a low CPI was the highest among these 3 indicators (low RVFAC; 0.622, low RVEF; 0.620, and low CPI; 0.665), which may have suggested the prediction of the primary endpoint using the CPI may not have been inferior to the CMR‐derived RVEF. The multivariate models were adjusted for the prevention, LV dysfunction, class 3 antiarrhythmic drugs, and CPI. In a multivariate analysis, a low CPI was an independent predictor of the composite endpoint defined as death and an initial appropriate ICD therapy (hazard ratio 13.5, 95% CI: 1.80–100.6 *p* = 0.011), and a class 3 antiarrhythmic drug was also an independent predictor. A low RVFAC was also an independent predictor in each multivariate model, which was adjusted for the prevention, LV dysfunction, class 3 antiarrhythmic drugs, and each RV function indicator. Among all parameters, low CPI had the highest hazard ratio. Moreover, LV dysfunction, defined as an LVEF < 35%, was not an independent predictor of death and an appropriate ICD therapy.

## 4 Discussion

This is the first study to compare multiple imaging modalities to evaluate right ventricular dysfunction to stratify the risk of death and life‐threatening arrhythmias. In the present study, we found that (1) RV dysfunction was significantly and independently associated with a worse prognosis, (2) the RVFAC, RVEF, and CPI all had good performance for stratifying the risk of SCD, whereas the CPI, which was the unique indicator among these, had the highest *c*‐statistic, and (3) the EPAP in patients with RV dysfunction was significantly higher than that in those with preserved RV function.

### 4.1 Prediction of Death and an Appropriate ICD Therapy

Previous studies have demonstrated the correlation between RV function and poor prognosis. A low RVEF measured on CMR has been identified as an independent predictor of all‐cause mortality in patients with nonischemic heart failure [14]. Similarly, a low RVFAC measured on TTE has also been associated with SCD in patients with a reduced LVEF [10]. According to a previous report, the tricuspid annular plane systolic excursion (TAPSE) showed a weaker correlation with RVEF (*r* = 0.63, *p* < 0.001), whereas RVFAC is more strongly correlated with RVEF (*r* = 0.81, *p* < 0.001) [15]. This difference is attributed to the fact that TAPSE measures only longitudinal contraction, reflecting movement in a single dimension, whereas RVFAC encompasses both longitudinal and short‐axis contractions. In the present study, the CPI has demonstrated the highest *c*‐statistics, suggesting its effectiveness as a strong competitor among indicators such as RVEF and RVFAC. In this study, LVEF was not identified as an independent predictor of the primary endpoint. This may be explained by the fact that 48% of patients underwent ICD implantation for secondary prevention and had preserved LV function at baseline. Furthermore, 58% of the patients who experienced the primary endpoint received ICDs for secondary prevention. Consequently, among patients with events, LVEF remained relatively high, potentially limiting its statistical power to emerge as an independent prognostic factor in the multivariable analysis. These results suggest that the observed independence of RVFAC from LVEF may reflect the specific features of the study population. Additionally, class 3 antiarrhythmic drug use was also identified as an independent predictor of the endpoint. This may be explained by the fact that patients receiving antiarrhythmic therapy at the time of implantation had a prior history of VT/VF, which likely contributed to the increased event rate.

### 4.2 RV Dysfunction and Sudden Cardiac Death

Generally, a myocardial infarction in the right ventricle typically leads to RV dysfunction and remodeling due to direct injury to the myocardium. Furthermore, chronic lung diseases can also lead to RV disorders by imposing a chronic afterload on the RV. Specifically, in patients with chronic obstructive pulmonary disease (COPD), there is a recognized increase in the risk of sudden cardiac death (SCD) due to hypoxia and RV overload from high pulmonary vascular resistance [16]. In the present study, we demonstrated that patients with RV dysfunction exhibited higher pulmonary artery pressure, although no association was found with right coronary artery lesions or organic lung diseases. Additionally, fibrotic remodeling, which is commonly associated with altered ventricular function in arrhythmogenic right ventricular cardiomyopathy (ARVC), involves fibro‐fatty replacement of the RV myocytes, leading to ventricular arrhythmias related to the RV [17]. Cardiac stresses such as pressure overload, inflammation, and aging, induce changes in the RV extracellular matrix and accumulate myofibroblasts [18], further contributing to RV dysfunction and ventricular arrhythmias that could lead to SCD, independent of left ventricular (LV) function.

Among patients classified as UVT, all patients demonstrated normal LV function and no structural abnormalities on CMR, cardiac computed tomography, or endomyocardial biopsy. In addition, coronary artery disease was ruled out by coronary angiography. However, several UVT patients had RV dysfunction despite preserved LV function, raising the possibility of early or subclinical cardiomyopathic processes. It is recognized that conditions such as cardiac sarcoidosis, myocarditis, or ARVC may remain undetectable in their early phases by conventional imaging or biopsy due to patchy myocardial involvement or mild inflammatory activity [19, 20]. These limitations suggest that UVT may reflect early disease not detected by current methods. Therefore, longitudinal follow‐up and consideration of advanced imaging or repeated assessment may be necessary for accurate etiologic diagnosis and risk stratification in this population.

### 4.3 Pulmonary Artery Pressure and RV Dysfunction

Melenovsky et al. reported that among patients with heart failure who have a preserved ejection fraction, RV dysfunction is associated with higher pulmonary arterial pressure (PAP), and RVFAC is significantly reduced compared to the controls even after accounting for the elevated PAP [21]. This suggests that the primary issue is an impairment of the RV contractility, rather than merely an afterload mismatch between the PAP and RV function, as the principal cause of the dysfunction.

Further analysis using a high‐fidelity conductance catheter revealed insights into RV‐pulmonary artery (PA) coupling, illustrating how PAP and volume dynamics interact. Notably, decreased RV contractility leads to RV‐PA uncoupling, which manifests as reduced stroke volume and increased systolic PAP [22, 23]. These findings indicate that in conditions where PA resistance and left heart pressure afterload are low, a high PAP still necessitates robust RV contractility. This relationship can be depicted through a linear correlation between these two parameters in a healthy population.

In this context, dividing RVFAC by the EPAP could reveal deviations from normal correlations, thus helping to uncover subtle RV dysfunctions. Consequently, a high CPI may signify an increased afterload. Therefore, the CPI emerges as a sensitive indicator, capable of predicting the need for appropriate ICD therapy and potential mortality risks. PAP estimation using IVC diameter and collapsibility is known to be prone to overestimation. To account for this, we performed an additional analysis using TRPG‐based estimation of PAP. In this alternative model, the contraction–pressure index was calculated as RVFAC divided by TRPG instead of PASP, and showed comparable predictive performance (AUC: 0.68, HR: 6.69, *c*‐statistic: 0.64). The IVC‐derived PASP (CPI) yielded slightly better overall discrimination and was therefore adopted for the primary analysis. Although ventricular leads may influence TR severity and TRPG, the type of ICD (transvenous vs. subcutaneous) was not associated with adverse outcomes in our cohort.

The utility of CPI as a marker lies in its ability to elucidate the relationship between RV function and pulmonary arterial pressure, providing a comprehensive assessment of cardiovascular function. By integrating the effects of both pulmonary and systemic pressures, CPI aids in identifying patients at risk of adverse outcomes from increased afterload, facilitating timely therapeutic interventions.

### 4.4 Multimodality Approach for the RV Function

Assessing the size and function of the right heart is crucial due to its unique anatomy and physiology, necessitating a multimodality approach. Methods include cardiac magnetic resonance imaging (CMR), transthoracic echocardiography (TTE), nuclear imaging, and cardiac computed tomography (CCT). TTE is non‐invasive, portable, widely available, and cost‐effective, making it a popular choice. Although CMR provides detailed and reproducible measurements, it is expensive, resource‐intensive, time‐consuming, and subject to several contraindications. CCT can also assess RV function and size, showing a good correlation with CMR; however, it tends to overestimate the RV ejection fraction (RVEF) by approximately 4.67% [24, 25]. Furthermore, TTE indicators are valuable for stratifying the risk of death and determining the need for appropriate implantable cardioverter‐defibrillator (ICD) therapy. The contraction pressure index (CPI), measured exclusively via TTE, is cost‐effective and predicts outcomes as accurately as the RVEF measured by CMR. Recent advancements in three‐dimensional echocardiography have enabled comparable quantification of RV function to that of CMR, offering another non‐invasive and economical option for cardiac assessment.

## 5 Limitations

There were significant limitations regarding the present study. First, this study was carried out in a retrospective, single‐center, and small cohort. Second, we did not analyze any other RV function measurements on TTE like the TAPSE or free‐wall strain. Third, the EPAP could not be measured in some cases due to a lack of TR or IVC data, so they were excluded from the CPI analysis. Finally, the tachyarrhythmia settings of the shock therapy and ATP therapy, as well as the bradycardiac therapy, differed in each patient.

## 6 Conclusion

The RV indicators of the RVFAC, RVEF, and CPI were associated with an increased incidence of death and an appropriate ICD therapy. The CPI seemed to provide a better stratification of the risk of death and an appropriate ICD therapy.

## Funding

The authors have nothing to report.

## Ethics Statement

This study was approved by the Research Ethics Committee of Chiba University (Reference number: M10039).

## Conflicts of Interest

The authors declare no conflicts of interest.
